# Characterization of a Polyamine Microsphere and Its Adsorption for Protein

**DOI:** 10.3390/ijms14010017

**Published:** 2012-12-20

**Authors:** Feng Wang, Pei Liu, Tingting Nie, Huixian Wei, Zhenggang Cui

**Affiliations:** 1 School of Chemical and Material Engineering, Jiangnan University, Wuxi 214122, China; E-Mails: liupei13579@163.com (P.L.); nietingting0128@163.com (T.N.); hxweiz@yahoo.com.cn (H.W.); cuizhenggang@hotmail.com (Z.C.); 2 The Key Laboratory of Food Colloids and Biotechnology of Ministry of Education, Jiangnan University, Wuxi 214122, China

**Keywords:** polyamine microsphere, characterization, selective adsorption, protein, lipase

## Abstract

A novel polyamine microsphere, prepared from the water-in-oil emulsion of polyethylenimine, was characterized. The investigation of scanning electron microscopy showed that the polyamine microsphere is a regular ball with a smooth surface. The diameter distribution of the microsphere is 0.37–4.29 μm. The isoelectric point of the microsphere is 10.6. The microsphere can adsorb proteins through the co-effect of electrostatic and hydrophobic interactions. Among the proteins tested, the highest value of adsorption of microsphere, 127.8 mg·g^−1^ microsphere, was obtained with lipase. In comparison with other proteins, the hydrophobic force is more important in promoting the adsorption of lipase. The microsphere can preferentially adsorb lipase from an even mixture of proteins. The optimum temperature and pH for the selective adsorption of lipase by the microsphere was 35 °C and pH 7.0.

## 1. Introduction

Microspheres have shown great potential for use as a controlled delivery carrier [1,2], catalyst [3], electronic material [4], *etc.* In the field of biotechnology, various functionalized microspheres have been applied for the separation, purification and immobilization of protein [5–8]. Protein adsorption on a solid surface is driven by the combinative forces of electrostatic, hydrophobic, hydrogen bonding, and specific chemical interactions [9,10]. Hence, the choice of the matrix material of microspheres is most important in designing application.

Polyethyleneimine (PEI) is a highly branched cationic polymer containing a high density of ionizable tertiary, secondary and primary amino groups. PEI can establish a nondistorting and very strong ionic interaction with protein [11], and has been used to increase protein loading on the solid surface [12]. Techniques of electrostatic adsorption [13], spray drying [14], covalent graft [12] and layer-by-layer assembly [15] have been developed to introduce PEI on surface of inorganic or organic solid sphere. Spray drying often is used as an encapsulation technique by the food [16] and pharmaceutical industry [17]. Murakami *et al.* made use of the technique to produce PEI-coated hydroxyapatite with various fractions of PEI [14]. The correlation between coating fraction and the retention time of protein or nucleotide in the chromatographic column was revealed. Although the PEI layer formed by spray drying is dehydrated, the layer is an unstable layer that gets released into the surrounding environment or solution due to the water solubility of PEI. Electrostatic adsorption is another important technique has been applied to form PEI layer on solid surface. Since PEI is a polycationic polymer, it can be electrostatically adsorbed on the negatively charged substrate to form a polyelectrolyte layer [18,19]. However, coatings that physically entrap PEI by electrostatic attraction in the absence of chemical bonds are structurally unstable and detached from the substrate easily. Unlike electrostatic adsorption, the grafting of PEI on the substrate covalently can form stable tethers, thereby avoiding amine leaching [20]. To graft PEI, the grafting agents such as γ-chloropropyl trimethoxysilane, chloroacetyl chloride and 3-glycidoxypropyltrimethoxysilane were used to offer epoxy group, aldehyde group and the structural segment of alkyl halide respectively, to react with amine groups on PEI macromolecule chains to form covalent bonds [12,20,21]. Similar to the graft of PEI by covalent bond, layer-by-layer (LBL) assembly via the alternate covalent reactions for constructing multilayer films on substrate can also offer the long-term structural stability. In the study by Hu *et al.* [22], the crosslinked multilayer films were formed through an alternate deposition of *p*-nitrophenyloxycarbonyl group-terminated hyperbranched polyether and PEI on aminolyzed quartz slide and silicon wafer based on the rapid reaction between *p*-nitrophenyloxycarbonyl group and primary amine. In another report by Xia *et al.* [23], PEI thin films were built up on aminosilanized glass surfaces via a layer-by-layer process of glutaraldehyde crosslinking. The chemically-crosslinked PEI films had a good long-term stability as compared with aminosilanized surfaces. However, the PEI loading on substrate by the covalent graft or the LBL assembly is limited by the surface area, the number of accessible active groups on substrate, and the complexity in synthesis conditions. In recent years, core removal method has been employed in obtaining PEI hollow particles. Sunintaboon *et al.* used dichloromethane to remove poly(methyl methacrylate) core of a poly(ethyleneimine)-*g-*poly( methyl methacrylate) copolymer for preparing hollow PEI particle [24]. MnCO_3_ microparticles coated with PEI layer have been reported to be used in the production of PEI microcapsules by the core removal method [25]. However, the chief drawbacks to the core removal method are the time-consuming procedure, thin shell and the possible structural changes of shell caused by removing the core with solvent.

The droplet of emulsion adopts spherical shape. Each droplet can be used as an independent nanoreactor under preservation of droplet size, droplet number, and concentration in each droplet [26]. When emulsion droplet is used as the vessel or template for polymerization, the size and shape of product are determined [27]. The polymerization using the emulsion droplet as template can also simplify procedure and facilitate the collection of product, e.g., the product can be separated from reaction mixture by centrifugation. In this study, we illustrate the structure characteristics of a novel polyamine microsphere prepared from PEI using inverse emulsion as the polymerization template. The adsorption capacity of the microsphere for various proteins was investigated.

## 2. Results and Discussion

### 2.1. Structure of the Polyamine Microsphere

In the inverse emulsion of PEI solution/liquid paraffin, the nonionic emulsifier, Span-80, adsorbed at the interface, kept the emulsion droplets separated by causing them to be repelled from one another through the steric stabilization mechanism. When added by a dropwise feeding mode, the glutaraldehyde solution dispersed into drops and coalesced with the emulsion droplets as the result of the process of interfacial free energy minimization. Then, glutaraldehyde molecules diffused from the interface to the inside of emulsion droplet to generate thermally and chemically stable crosslinks with the amine groups of PEI by the Schiff reaction to form polyamine microsphere.

**Figure 1:**
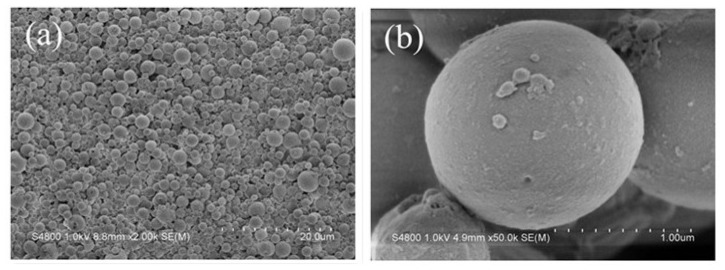

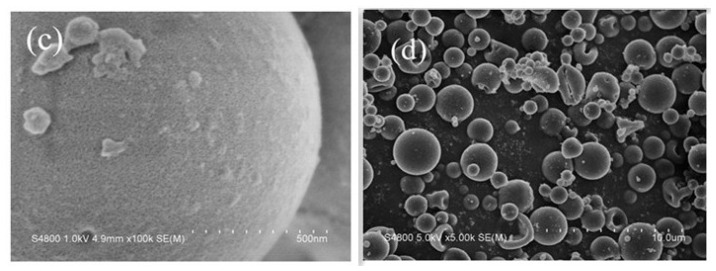
SEM images of polyamine microsphere: (**a**) Image of an aggregation of microsphere; (**b**) Magnification of an intact microsphere; (**c**) Zoom-in image of the outer surface of microsphere; (**d**) Image of the microsphere obtained by reducing the adding amount of glutaraldehyde by half.

Image of an aggregation of the polyamine microsphere was observed by scanning electron microscopy (SEM) and the result is displayed in Figure 1a. The image indicates that the microsphere is a kind of polydisperse microsphere. The magnified images of a single perfect microsphere in Figure 1b,c show that the microsphere is regular in shape. The number of collapsed or broken microsphere was found increased by decreasing the addition amount of glutaraldehyde (Figure 1d). The reason for this may be that the strength of shell of microsphere was insufficient to sustain the hollow structure after removing the core material of water.

**Figure 2:**
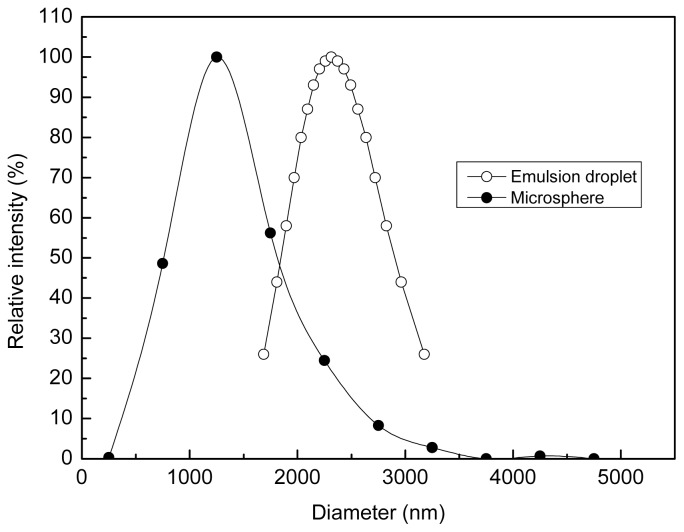
Diameter distribution of the polyamine microsphere and the emulsion droplet used as the template for crosslinking.

The diameter distribution of the polyamine microspheres was found to be in the range of 0.37–4.29 μm, and the average diameter of the microsphere was 1.44 μm, which is less than that of the emulsion droplet used as the template (Figure 2). The reason accounts for the change in diameter may be that the polycondensation of PEI makes the arrangement of molecules become closer, reducing the occupied volume.

**Figure 3:**
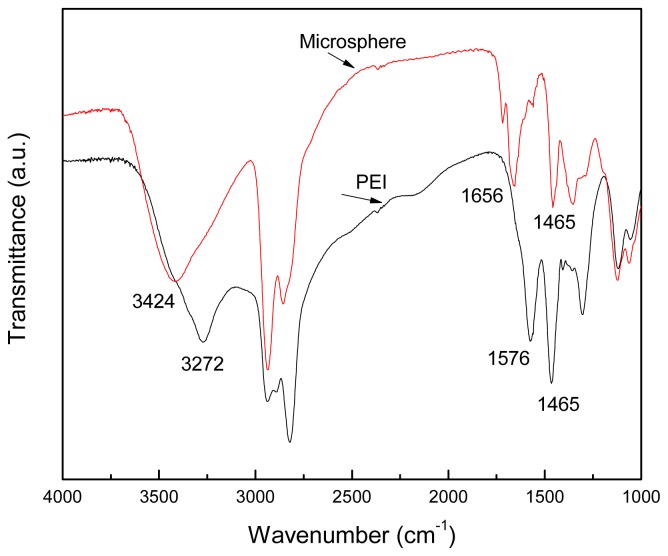
The FTIR spectra of PEI and the polyamine microsphere.

The chemical structure of PEI and the polyamine microsphere were identified with Fourier transformation infrared spectroscopy (FITR) and the results are shown in Figure 3. Characteristic peaks of PEI at 3272 cm^−1^ (–N–H stretching), 2940–2830 cm^−1^ (–C–H stretching), 1576 cm^−1^ (–N–H bending), 1465 cm^−1^ (–C–H bending) and 1350–1000 cm^−1^ (–C–N stretching) can be found in the spectrum of polyamine microsphere. Unlike PEI, the FITR spectrum of polyamine microsphere displays a distinct peak at 1656 cm^−1^, which is the stretching band of –C=N, indicating the Schiff reaction between the amine groups of PEI and the aldehyde groups of glutaraldehyde. The peak of stretching vibration of –N–H appears is at 3272 cm^−1^ in the spectrum of PEI, which transfers to 3424 cm^−1^ in the spectrum of polyamine microsphere. It should be noted that the presence of water molecules also result in the appearance of an absorption peak at 3424 cm^−1^.

### 2.2. Thermalstability and Zeta Potential of the Polyamine Microsphere

**Figure 4:**
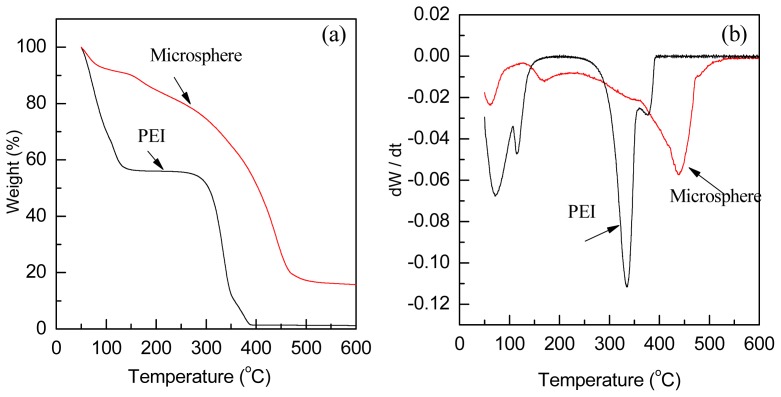
TG (**a**) and DTG (**b**) thermograms of PEI and polyamine microsphere.

Thermalstability of PEI and the polyamine microsphere were evaluated by thermogravimetry (TG) and derivative thermogravimetry (DTG) (Figure 4). According to the DTG curves (Figure 4b), both PEI and the polyamine microsphere present an initial mass loss at the range from 50 °C to 100 °C, due to the loss of physically adsorbed CO_2_ [28]. For PEI, the TG curve shows a two-stage weight loss between 50 °C and 600 °C (Figure 4a). The weight loss in the first stage, from 50 °C to 161.5 °C, was 44%. The DTG curve peak of PEI at the range from 106 °C to 161.5 °C is corresponding to the evaporation of water from the solution of PEI. At the stage between 253 °C and 397.6 °C, the endothermic peak of DTG curve is ascribed to the decomposition of PEI. The weight loss at this stage demonstrates that PEI decomposed completely with a decomposition residue of about 1.4%. In the case of the polyamine microsphere, the total weight loss was 83.2% from 50 °C to 550 °C (Figure 4a). The DTG curve of microsphere shows an obvious peak at the range from 132.5 °C to 209 °C, which is attributable to the decomposition or removal of residual Span-80 or glutaraldehyde. The weight loss in this range was 6.6%. As displayed in the DTG curve, the decomposition rate of the microsphere reaches the extremum at 440 °C. Therefore, the polyamine microsphere is more stable than PEI.

**Figure 5:**
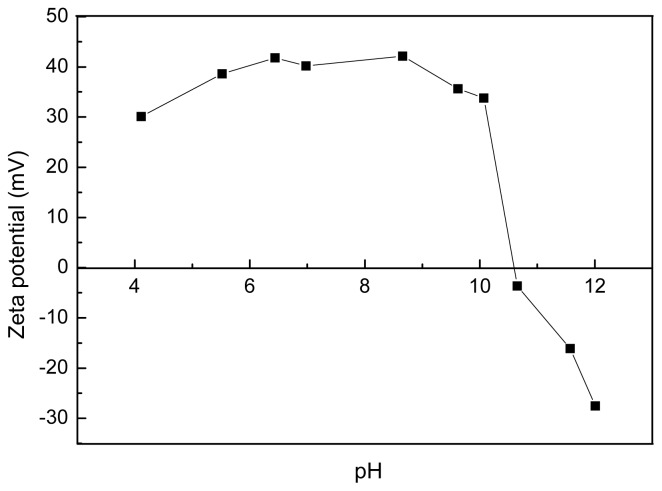
Zeta potentials of 1 wt% polyamine microsphere ultrasonically dispersed in water of different pH.

Analysis of the surface charge of polyamine microsphere was carried out according to the results obtained by Zeta potential measurement. The plot of Zeta potential of the microsphere as the function of pH is presented in Figure 5. The isoelectric point (pI) of the microsphere is 10.6. Hence, the microsphere is positively charged when pH < 10.6, owing to the protonation on amine groups. The maximal potential is around +40 mV between pH 6.5 and pH 8.5. The Zeta potential decreased and become negative when pH > 10.6. In the range of pH investigated, the lowest value of Zeta potential was found to be −27.54 mV at pH 12.

### 2.3. Protein Adsorption of the Microsphere

**Figure 6:**
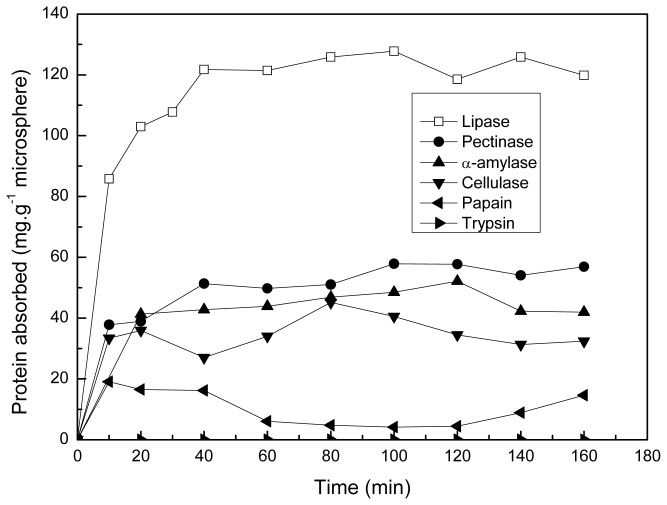
Dynamic curves of proteins adsorption on the polyamine microsphere (pH 7.0, 25 °C).

The adsorptions of various proteins by the polyamine microsphere at pH 7.0 and 25 °C are demonstrated in Figure 6. Since the isoelectric point of the microsphere is 10.6, the microsphere is positively charge at pH 7.0. It was found, except for that of lipase and α-amylase adsorption, the maximal value of proteins adsorption decreased with the increase of pI of proteins.

According to the values of isoelectric points (pI), the proteins tested can be divided in to two classes. In the first class, there are cellulase (pI 4.75), lipase (pI 4.9), pectinase (pI 3.6), α-amylase (pI 6.7). These proteins are negatively charged at pH 7.0. Hence, these proteins can be adsorbed by the positively charged microsphere through electrostatic interaction at pH 7.0. Although the pI value of lipase is close to that of cellulase, the adsorption of lipase on the microsphere (127.8 mg·g^−1^ microsphere) is highest.

The second class consists of trypsin (pI 11.0) and papain (pI 8.75). These two proteins are positively charged at pH 7.0. The electrostatic repulsion between the microsphere and the proteins charged with like charges do not favor the adsorption of trypsin and papain on the microsphere. Hence, the adsorption of trypsin and papain are lower than that of the other proteins tested at pH 7.0.

**Figure 7:**
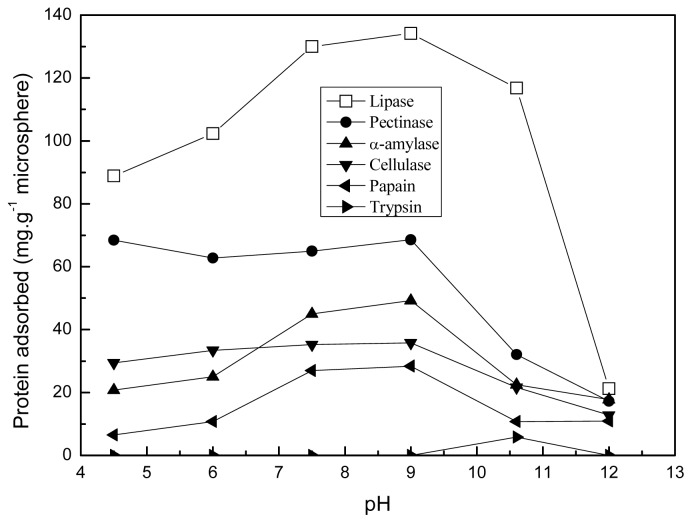
The protein adsorption of the polyamine microsphere at various pH and at 25 °C.

Figure 7 demonstrates the changes of protein adsorption at different pH (25 °C). When the pH of solution was adjusted between the pI of protein and the pI of microsphere, the protein adsorption was relative higher. In this case, the negatively charged proteins are electrostatically attracted by the positively charged microsphere. When the pH value of solution was 10.6 (the pI of microsphere), the surface potential of polyamine microsphere was zero. The electrostatic interaction between the microsphere and protein was inhibited in this situation, while the hydrophobic force became the main driving force promoting the adsorption of protein. According to the results showed in Figure 7, the adsorption of lipase, pectinase, α-amylase, cellulase, papain is reduced by 13%, 53.2%, 54.3%, 39.7%, 62% respectively at 10.6. Hence, the hydrophobic interaction is more important for the adsorption of lipase on the polyamine microsphere in comparison with that of the other proteins tested. Lipase prefers to adsorb on hydrophobic supports, involving in the adsorption of the hydrophobic areas surrounding the active center [29–31]. During the crosslinking of PEI with glutaraldehyde, the hydrocarbon segments in the molecular chains of PEI and glutaraldehyde intertwined to form hydrophobic patches on the polyamine microsphere. The hydrophobic patches can contribute to the adsorption of proteins by hydrophobic interaction.

It was found trypsincan not be adsorbed by the microsphere in the tested pH conditions except for pH 7.0. The reason is that trypsin (pI 11.0) and the microsphere (pI 10.6) were charged like charges except that in the case of pH 10.6, which hindered the adsorption of trypsin by electrostatic repulsion. The adsorption of trysin at pH 10.6 is due to the interaction driven by hydrophobic force.

### 2.4. Selective Adsorption of Lipase onto the Polyamine Microsphere

**Figure 8:**
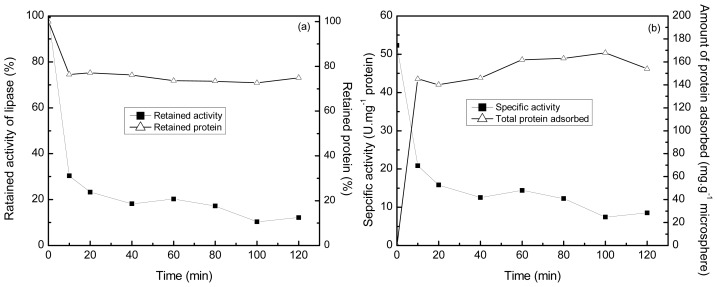
Time profiles of retained activity of lipase and retained protein during adsorption (**a**) specific activity of lipase in solution and the total amount of protein adsorbed by the microsphere (**b**) pH 7.0, 25 °C.

**Figure 9:**
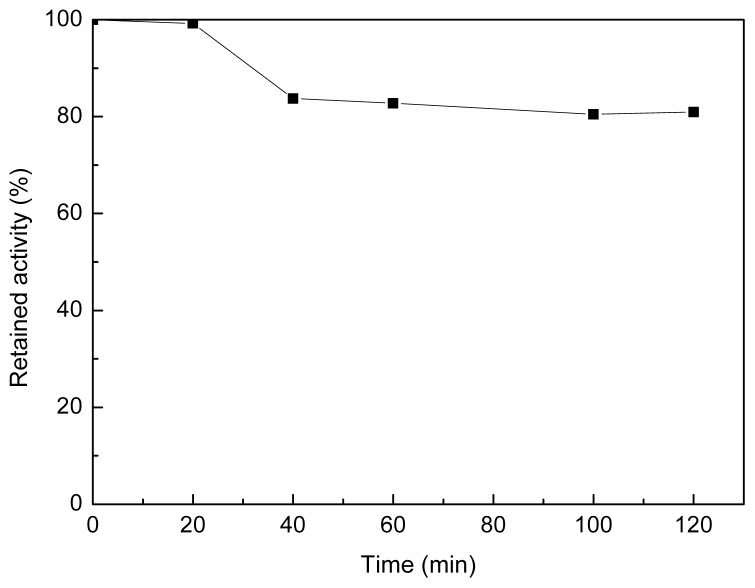
Stability of lipase in the even mixture of proteins at 25 °C and pH 7.0.

The selectivity of the polyamine microsphere for the adsorption of lipase was investigated in an even mixture of lipase, papain, cellulase and α-amylase prepared at 25 °C and pH 7.0. The initial concentration of protein in solution was 3 mg·mL^−1^. As the results demonstrated in Figure 8, the amount of protein adsorbed by the microsphere arrived at the highest value of 161.9 mg·g^−1^ microsphere in one hour of incubation. The specific activity of lipase in solution decreased from the initial value of 52.3 U·mg^−1^ to 14.4 U·mg^−1^ in the same period. The further decrease in the specific activity of lipase after 1 h of incubation is ascribed to the deactivation of lipase since the variation of protein content in solution was negligible in the duration. In one hour of incubation, the total activity of lipase in solution was reduced by about 80%, while the content of protein was reduced by 26.46%. The investigation of the stability of lipase revealed that the residual activity of lipase in the mixture was about 80% of its initial activity in two hours of incubation at 25 °C and pH 7.0 (Figure 9). Hence, taking the deactivation ratio of lipase into account, the microsphere can selectively adsorb lipase from the even mixture of proteins tested.

### 2.5. Effect of pH and Temperature on the Adsorption

**Figure 10:**
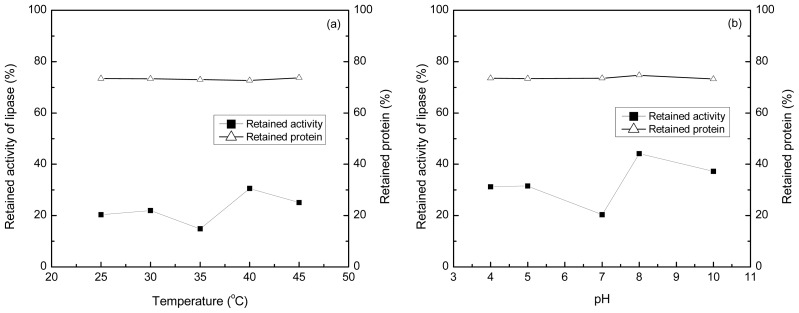
Influences of various temperatures and pH on the maximal values of protein adsorption on the microsphere and the retained activity of lipase.

The profiles of maximal amount of protein adsorption and retained lipase activity in solution related to temperature and pH were constructed at 25–45 °C and pH 4.0–10.0 (Figure 10). From the experimental results, it can be found that the maximal amount of protein adsorbed by the microsphere was not influenced by the variation in temperature and pH. The lowest value of retained activity of lipase in solution was 14.8% and 20.4% of its initial activity at 35 °C and pH 7.0 respectively. With the increase of temperature and pH, the retained activity of lipase elevated. Hence, the polyamine microsphere can preferentially adsorb lipase from the even mixture of lipase, pectinase, cellulase and α-amylase.

## 3. Experimental Section

### 3.1. Materials

Polyethyleneimine (Mw = 70,000 Da; 50 wt% solution in water), bovine pancreas trypsin (EC 3.4.21.4; pI 11.0), cellulase from *Trichoderma viride* (EC 3.2.1.4; pI 4.75), pocine pancreas lipase (EC 3.1.1.3; pI 4.9), pectinase from *Aspergillus niger* (EC 3.2.1.15; pI 3.6), papain from papaya latex (EC 3.4.22.2; pI 8.75), α-amylase from porcine pancreas (EC 3.2.1.1; pI 6.7) were purchased from Sigma-Aldrich Shanghai Trading Co. Ltd., Shanghai, China. The proteins were purified by using a desalting column (Econo-Pac 10 DG, Bio-Rad, Foster City, CA, USA) in advance [12]. Glutaraldehyde solution (25 wt %), liquid paraffin, Span-80 was obtained from Sinopharm Chemical Reagent Co., Ltd., Shanghai, China. Other reagents are of analytical grade and used as received.

### 3.2. Preparation of Polyamine Microsphere in Inverse Emulsion

In a glass bottle of dimensions 7.5 cm (h) by 2.5 cm (d), a mixture of PEI solution (20 wt%), liquid paraffin and Span-80 was homogenized at 3000 rpm for 2 min using a XHF-D dispersator (Ningbo Xinzhi, Zhejiang, China) fitted with a dispersing tool of outer diameter equals to 1.4 cm. The volume ratio between PEI solution and liquid paraffin was 8:12. The amount of Span-80 used was 0.04 g·mL^−1^ relative to the oil phase. The water in oil emulsion formed in this way was transferred to a 100 mL round-bottomed flask. Under the condition of moderate magnetic stirring, glutaraldehyde solution was fed into the emulsion in a dropwise mode through a latex tube (0.8 mm of the inside diameter) with a syringe pump at the speed of 0.2 mL·min^−1^. The amount ratio of glutaraldehyde to PEI was 1:1.48 × 10^−5^ (mol:mol). The crosslinking reaction was conducted for 15 h at the room temperature. Finally, the polyamine microsphere was collected by centrifugation and rinsed with diethyl ether, isopropyl alcohol and deionized water successively, and dried in vacuum.

### 3.3. The Adsorption of Protein

In a 50 mL flask with stopper, 0.05 g polyamine microsphere was incubated with 10 mL of 1 mg·mL^−1^ protein solution at 25 °C on a rotary shaker with an agitation speed of 200 rpm. At regular intervals, samples were taken from the flask and filtered through 0.22 μm millipore filter membrane. The amount of protein absorbed was estimated based on the reduction of the amount of protein in the solution of adsorption. In the experiment for investigating the adsorption selectivity of the polyamine microsphere, an even mixture of lipase, papain, cellulase and α-amylase was prepared to attain a total amount of protein 3 mg·mL^−1^. The influence of temperature and pH on the adsorption was investigated in the range of 25–45 °C and 4.0–6.0 respectively. The pH of above solutions was adjusted by 0.1 M HCl or 0.1 M NaOH solution. All experiments were performed at least three times.

### 3.4. Determination of Protein Content and the Activity of Lipase

The protein content in solution was measured by the Lowry method [32].

The hydrolytic activity of lipase solution was determined by titrimetric assay according to an olive oil emulsion method with some modifications [33]. 50 mL of Olive oil was emulsified in 150 mL distilled water containing 4 wt% PVA using the XHF-D dispersator at 3000 rpm for 6 min. The assay mixture consisted of 4 mL emulsion, 5 mL phosphate buffer (0.025 mol·L^−1^, pH 7.5) and 1 mL sample. The oil hydrolysis was carried out at 40 °C for 15 min, and then stopped by adding 15 mL ethanol. Enzyme activity was determined by titration of the fatty acid released with 50 mmol·L^−1^ NaOH solution. One activity unit of lipase was defined as the amount of enzyme, which released 1 μmol of fatty acid per minute under the assay condition. The specific activity (U·mg^−1^ of protein) was calculated by dividing the enzyme activity by the protein content.

### 3.5. Characterization of the Polyamine Microsphere

Optical microscope imaging was performed using a VHX-1000 Optical microscopy (Keyence corporation, Osaka, Japan) equipped with a digital camera (Fujifilm, Tokyo, Japan). Morphological structure of microsphere was examined by a S-4800 scanning electron microscope (Hitachi Ltd., Tokyo, Japan). Fourier transformation infrared spectra (FTIR) were obtained using a Tensor 27 spectrometer (Bruker corporation, Ettlingen, Germany). Thermogravimetry (TG) and derivative thermogravimetry (DTG) analysis were performed on a TGA/SDTA851e thermogravimetric analyzer (Mettler-Toledo International Inc., Zürich, Switzerland). The diameter distribution of emulsion droplets and the Zeta potential of microsphere dispersed ultrasonically in the water of different pH were measured using a ZetaPlus zeta potential analyzer (Brookhaven instruments corporation, New York, NY, USA) at 25 °C. The diameter distribution of microsphere was determined by the method described by Cohen *et al.* [34].

## 4. Conclusions

In this study, the polyamine microsphere prepared using the inverse emulsion of polyethyleneimine as the template was characterized by SEM, FTIR and TG analysis, and Zeta potential determination. The microsphere can adsorb protein through electrostatic interaction and hydrophobic interaction. The effect of hydrophobic force on the adsorption of lipase was greater than that of the other proteins tested. The adsorption of lipase by the microsphere was significantly higher than that of the other proteins. The adsorption selectivity of the polyamine microsphere for lipase can be performed at 35 °C and neutral pH. The polyamine microsphere has a potential application in the environmental protection, separation chromatography for biomaterials and enzyme technology.
